# Joint Association of Sedentary Time and Physical Activity with Metabolic Syndrome Among Korean Adults: A Cross-Sectional Analysis of the 2024 Korea National Health and Nutrition Examination Survey

**DOI:** 10.3390/healthcare14193208

**Published:** 2026-09-28

**Authors:** Kyong Sil Park

**Affiliations:** Department of Nursing, Chungwoon University, Hongseong 32244, Republic of Korea; ks_park@chungwoon.ac.kr

**Keywords:** sedentary behavior, physical activity, metabolic syndrome, Korea national health and nutrition examination survey, Republic of Korea

## Abstract

**Highlights:**

**What are the main findings?**

- Participants with high sedentary time who did not meet the physical activity recommendation had higher odds of metabolic syndrome than the low sedentary time/meeting recommendation group after adjustment for sociodemographic factors and health-related behaviors.
- No statistically significant multiplicative or additive interaction between sedentary time and physical activity was observed. In sensitivity analysis, longer sedentary time assessed continuously was associated with higher odds of metabolic syndrome.

**What are the implications of the main findings?**

- Sedentary time and physical activity should be considered jointly when assessing metabolic health, but the present findings do not provide evidence that physical activity modifies or offsets the association between sedentary time and metabolic syndrome.
- Prospective studies using more detailed or objective measures of sedentary time and physical activity are needed to clarify dose–response relationships and their combined associations with metabolic health.

**Abstract:**

Background/Objectives: This study examined the joint association of sedentary time and physical activity with metabolic syndrome among Korean adults and whether physical activity modified this association. Methods: This cross-sectional study analyzed 2024 Korea National Health and Nutrition Examination Survey data from 3075 adults aged ≥19 years. Participants were classified into four groups by sedentary time and physical activity recommendation status. Complex-sample logistic regression was performed, with additional analyses of independent associations, interactions, and sensitivity analyses. Results: Compared with the low sedentary time/meeting group, the high sedentary time/not meeting group had higher odds of metabolic syndrome after adjustment for sociodemographic and health-related factors (OR = 1.38, 95% CI: 1.07–1.78; *p* = 0.013). No significant multiplicative interaction was observed (interaction OR = 1.08, 95% CI: 0.74–1.57; *p* = 0.681), and additive interaction indices were also nonsignificant. Each additional hour/day of sedentary time was associated with 4% higher odds of metabolic syndrome (OR = 1.04, 95% CI: 1.01–1.07; *p* = 0.011). After additional BMI adjustment, the association for the high sedentary time/not meeting group was not statistically significant (OR = 1.34, 95% CI: 0.98–1.83; *p* = 0.065). Conclusions: High sedentary time combined with not meeting the physical activity recommendation was associated with higher odds of metabolic syndrome. However, no significant multiplicative or additive interaction was identified. These findings support joint consideration of sedentary time and physical activity without implying that physical activity offsets the association of prolonged sedentary time with metabolic syndrome.

## 1. Introduction

Metabolic syndrome refers to the coexistence of several cardiometabolic abnormalities, including central obesity, hypertension, lipid abnormalities, and disturbances in glucose regulation, and is linked to a higher likelihood of developing cardiovascular disease and type 2 diabetes mellitus [1,2]. Among U.S. adults, the prevalence of metabolic syndrome was estimated at 34.7% during 2011–2016 [3]. In Korea, its prevalence among adults was reported to be 24.9% during 2019–2021, indicating a substantial national burden [4]. Metabolic syndrome is therefore an important public health concern because it contributes to increased healthcare utilization and costs associated with chronic disease management and may adversely affect work productivity [5]. The high prevalence of metabolic syndrome may be partly related to lifestyle changes associated with urbanization, expanding digital environments, and increasing time spent in sedentary work and leisure activities [6,7]. In particular, insufficient physical activity and prolonged sedentary behavior are potentially modifiable behavioral factors associated with adverse metabolic outcomes [8,9].

Prolonged sedentary behavior and insufficient physical activity may decrease skeletal muscle contractions and overall energy expenditure, potentially leading to impaired insulin sensitivity and abnormalities in glucose and lipid metabolism [7,10]. Sedentary behavior has also been associated with poorer metabolic health even after accounting for physical activity levels [6]. In contrast, regular physical activity may support metabolic health by improving insulin sensitivity, mitochondrial function, glucose regulation, and inflammatory profiles [11]. These findings support considering sedentary behavior and physical activity as distinct but interrelated movement behaviors when examining their associations with metabolic syndrome. Because individuals with similar sedentary time may differ substantially in their physical activity levels, examining each behavior separately may not fully characterize their combined relationship with metabolic health.

Although sedentary behavior and physical activity have each been extensively studied, they have often been examined separately, and evidence regarding their combined association with metabolic syndrome remains limited [12,13]. Previous studies examining joint patterns of sedentary time and physical activity have suggested that cardiometabolic outcomes may differ across combinations of these behaviors [12,14,15]. However, relatively few studies have formally evaluated whether physical activity modifies the association between sedentary time and metabolic syndrome using multiplicative or additive interaction measures. Moreover, few studies have examined combinations of sedentary time and physical activity using recent, nationally representative data from Korean adults. Examining these behaviors jointly and formally assessing their interaction may therefore provide a more comprehensive understanding of the behavioral patterns associated with metabolic syndrome.

The Korea National Health and Nutrition Examination Survey (KNHANES) is a nationally representative, population-based survey that includes self-reported measures of sedentary time and physical activity and clinically measured components of metabolic syndrome [13]. It therefore provides an appropriate data source for examining the joint association of these movement behaviors with metabolic health among Korean adults. Using data from the 2024 KNHANES, this study aimed to examine the association between four sedentary time–physical activity combinations and metabolic syndrome among adults aged 19 years and older. Additionally, we examined the independent associations of sedentary time and physical activity with metabolic syndrome and assessed whether physical activity modified the association between sedentary time and metabolic syndrome on multiplicative and additive scales.

## 2. Materials and Methods

### 2.1. Study Design and Data Source

This cross-sectional study analyzed data from the 2024 KNHANES, a nationally representative, population-based health survey conducted by the Korea Disease Control and Prevention Agency. The KNHANES uses a complex, stratified, multistage probability sampling design and consists of a health interview, a health examination, and a nutrition survey. This study examined the joint and independent associations of sedentary time and physical activity with metabolic syndrome and evaluated their interaction among Korean adults.

### 2.2. Study Participants and Data Selection

Of the 6997 participants in the 2024 KNHANES, 964 individuals aged younger than 19 years were excluded, leaving 6033 adults aged 19 years or older. To reduce potential reverse causation due to disease-related limitations in physical activity, we excluded participants who reported a history of cancer, myocardial infarction, angina pectoris, or stroke (*n* = 576). We then excluded participants with missing data on sedentary time or physical activity (*n* = 603), one or more components required to define metabolic syndrome—waist circumference, blood pressure, fasting plasma glucose, triglycerides, or high-density lipoprotein cholesterol—(*n* = 264), or covariates included in the primary multivariable models (*n* = 1515). The final analytic sample comprised 3075 participants. For the sensitivity analysis additionally adjusted for BMI, 27 participants with missing BMI data were further excluded, resulting in an analytic sample of 3048. The participant selection process is presented in Figure 1.

### 2.3. Ethical Considerations

This study used publicly available, de-identified data from the 2024 KNHANES. The study protocol was reviewed by the Institutional Review Board of Chungwoon University and was determined to be exempt from review (IRB exemption No. 1041566-202603-HR-001-01). All KNHANES participants provided informed consent during the original data collection, and no additional informed consent was required for this secondary analysis.

### 2.4. Measurement of Variables

#### 2.4.1. Sedentary Time

Sedentary time was assessed using a self-reported KNHANES health interview item asking participants how much time they usually spent sitting or lying down during a typical day, excluding sleep, across occupational, domestic, transportation, social, and leisure contexts. This item is derived from the sedentary behavior component of the Korean version of the Global Physical Activity Questionnaire (K-GPAQ). Previous validation of the K-GPAQ among Korean adults demonstrated acceptable test–retest reliability for sedentary behavior (Spearman’s r = 0.65), whereas criterion validity compared with accelerometer-measured sedentary behavior was relatively low (r = 0.18), suggesting potential measurement error in self-reported sedentary time [16].

Responses were recorded in hours and minutes and converted to hours per day. Participants were classified as having low sedentary time (≤8 h/day) or high sedentary time (>8 h/day), based on the threshold recommended in the Canadian 24-h Movement Guidelines for Adults [17]. These guidelines recommend limiting sedentary time to ≤8 h/day; therefore, the 8 h threshold was used as a guideline-based behavioral benchmark rather than as a diagnostic or disease-specific cutoff for metabolic syndrome [17]. To evaluate the robustness of the findings and address potential information loss resulting from dichotomization, sedentary time was additionally analyzed as a continuous variable per 1 h/day increase in a sensitivity analysis.

#### 2.4.2. Physical Activity

Physical activity was assessed using self-reported Global Physical Activity Questionnaire items included in the KNHANES health interview. Previous validation of the K-GPAQ among Korean adults showed that test–retest reliability varied by physical activity domain (Spearman’s ρ = 0.27–0.47 for work-related physical activity and 0.53–0.70 for recreational physical activity), while criterion validity for total physical activity compared with accelerometer measurements was weak but statistically significant (r = 0.34, *p* < 0.01) [16]. Participants reported the weekly frequency and daily duration of activities performed continuously for at least 10 min during work, transportation, and leisure. Weekly vigorous-intensity activity comprised work- and leisure-related vigorous activity, whereas weekly moderate-intensity activity comprised work- and leisure-related moderate activity and walking or cycling for transportation. In accordance with the World Health Organization physical activity recommendations [18], participants were classified as meeting the recommendation if they performed ≥150 min/week of moderate-intensity activity, ≥75 min/week of vigorous-intensity activity, or an equivalent combination of moderate- and vigorous-intensity activity. Participants who did not meet any of these criteria were classified as not meeting the recommendation. Although the 2020 WHO guidelines no longer require physical activity to be accumulated in bouts of at least 10 min, the 2024 KNHANES questionnaire retained the ≥10 min bout criterion; therefore, this criterion reflects the structure of the source questionnaire rather than an additional restriction imposed in the present analysis [18]. To address potential information loss resulting from dichotomizing physical activity as meeting versus not meeting the recommendation, sensitivity analyses additionally examined total moderate-to-vigorous physical activity (MVPA) time, calculated as the sum of weekly moderate- and vigorous-intensity activity minutes, as a continuous variable per 150 min/week increase and in four categories (0, 1–149, 150–299, and ≥300 min/week).

#### 2.4.3. Joint Categories of Sedentary Time and Physical Activity

Sedentary time and physical activity were cross-classified to create four mutually exclusive categories: (1) low sedentary time (≤8 h/day) and meeting the physical activity recommendation; (2) low sedentary time and not meeting the recommendation; (3) high sedentary time (>8 h/day) and meeting the recommendation; and (4) high sedentary time and not meeting the recommendation. The low sedentary time/meeting the recommendation category was used as the reference group. For interaction analyses, high sedentary time and not meeting the physical activity recommendation were coded as the respective exposure categories, with low sedentary time and meeting the recommendation serving as the joint reference condition.

#### 2.4.4. Metabolic Syndrome

Metabolic syndrome was defined according to the modified NCEP ATP III criteria [1], incorporating the revised fasting plasma glucose threshold and Korean-specific waist circumference cutoffs [19]. Participants were classified as having metabolic syndrome if they met at least three of the following five components: (1) abdominal obesity, defined as a waist circumference of ≥90 cm in men or ≥85 cm in women; (2) elevated triglycerides, defined as a triglyceride level of ≥150 mg/dL; (3) reduced high-density lipoprotein cholesterol (HDL-C), defined as an HDL-C level of <40 mg/dL in men or <50 mg/dL in women; (4) elevated blood pressure, defined as systolic blood pressure of ≥130 mmHg, diastolic blood pressure of ≥85 mmHg, or current use of antihypertensive medication; and (5) elevated fasting glucose, defined as fasting plasma glucose of ≥100 mg/dL or current use of glucose-lowering medication, including insulin.

### 2.5. Covariates

Covariates were selected a priori based on previous studies [20,21] and their potential associations with sedentary time, physical activity, and metabolic syndrome. Sociodemographic and socioeconomic covariates included age, sex, education level, and household income. Age was included as a continuous variable. Education level was categorized according to the highest level of education attained, and household income was classified into quartiles. Health-related behavioral covariates included smoking status, alcohol consumption, and average sleep duration. Smoking status and alcohol consumption were dichotomized as ever versus never based on self-reported lifetime experience. Average daily sleep duration was calculated as a weighted weekly average using the formula [(weekday sleep duration × 5) + (weekend sleep duration × 2)]/7. Body mass index (BMI) was calculated as measured weight in kilograms divided by the square of height in meters (kg/m^2^). BMI was not included as a conventional confounder in the primary adjusted model because it is closely related to waist circumference, which is a diagnostic component of metabolic syndrome, and may also lie on the pathway linking sedentary time and physical activity to metabolic syndrome. Therefore, Model 4 was considered the primary adjusted model, whereas additional adjustment for BMI was performed only as a sensitivity analysis to assess the robustness of the estimates.

### 2.6. Statistical Analysis

All analyses accounted for the complex sampling design of the KNHANES by incorporating sampling weights, stratification variables, and primary sampling units. Continuous variables are presented as weighted means with standard errors and were compared using complex-sample general linear models, whereas categorical variables are presented as unweighted frequencies with weighted percentages and were compared using Rao–Scott adjusted chi-square tests.

Complex-sample logistic regression was used to estimate odds ratios (ORs) and 95% confidence intervals (CIs) for the associations between the joint sedentary time–physical activity categories and metabolic syndrome. Model 1 was unadjusted. Model 2 was adjusted for age and sex; Model 3 was additionally adjusted for education level and household income; and Model 4 was further adjusted for smoking status, alcohol consumption, and average sleep duration. Model 4 was considered the primary adjusted model. Model 5 additionally included BMI and was treated as a sensitivity analysis because of the potential for over-adjustment.

To examine the independent associations of sedentary time and physical activity with metabolic syndrome, sedentary time and physical activity were entered simultaneously into the multivariable logistic regression model, together with the covariates included in Model 4. Multiplicative interaction was assessed by including a sedentary time × physical activity product term in the fully adjusted logistic regression model. Effect modification was further explored by estimating the association between sedentary time and metabolic syndrome separately according to physical activity recommendation status and by directly comparing the two high-sedentary-time groups according to physical activity status.

Additive interaction was evaluated using the relative excess risk due to interaction (RERI), attributable proportion due to interaction (AP), and synergy index (SI). Because metabolic syndrome was relatively common in the study population, adjusted prevalence ratios were estimated using survey-weighted modified Poisson regression and were used to calculate the additive interaction indices and their 95% CIs. The 95% CIs for RERI and AP were estimated using the delta method, whereas the 95% CI for SI was estimated on the log scale using the delta method and then exponentiated.

Sensitivity analyses were performed by modeling sedentary time continuously per 1 h/day increase and physical activity continuously per 150 min/week increase. Physical activity was also categorized as 0, 1–149, 150–299, and ≥300 min/week. An additional sensitivity model included BMI. To assess potential selection bias related to sample exclusion, characteristics of participants included in and excluded from the final analytic sample were compared using complex-sample procedures. Primary analyses were based on complete cases, while available observations were used for each characteristic in the included-versus-excluded comparison. No formal multicollinearity diagnostics, such as variance inflation factors or tolerance statistics, were performed for the multivariable regression models. All statistical tests were two-sided, and *p* < 0.05 was considered statistically significant. Analyses were performed using IBM SPSS Statistics for Windows, version 26.0 (IBM Corp., Armonk, NY, USA).

## 3. Results

### 3.1. General Characteristics of Participants According to Joint Sedentary Time–Physical Activity Categories

The weighted mean age of the study population was 49.0 years, and the weighted mean age differed significantly across the four joint sedentary time–physical activity categories (*p* < 0.001). Participants in the low sedentary time/not meeting the physical activity recommendation category had the highest mean age (56.4 years), whereas those in the high sedentary time/meeting the recommendation category had the lowest mean age (41.6 years).

Sex distribution also differed significantly across the categories (*p* = 0.007), with the highest weighted proportion of men observed in the high sedentary time/meeting the recommendation category (57.1%). Educational attainment and household income differed significantly across the categories (both *p* < 0.001). The high sedentary time/meeting the recommendation category had the highest weighted proportions of participants with a college education or higher and those in the highest household income quartile.

Smoking status did not differ significantly across the categories (*p* = 0.960). In contrast, alcohol consumption status differed significantly (*p* < 0.001), with higher weighted proportions of ever-drinkers in the two categories that met the physical activity recommendation. Mean sleep duration differed significantly across the categories (*p* = 0.013), with higher mean values in the low sedentary time categories. BMI did not differ significantly across the categories (*p* = 0.440) (Table 1).

### 3.2. Prevalence of Metabolic Syndrome and Its Components According to Joint Sedentary Time–Physical Activity Categories

The weighted prevalence of metabolic syndrome differed significantly across the four joint sedentary time–physical activity categories (*p* < 0.001). The prevalence was higher in the categories that did not meet the physical activity recommendation—30.4% in the low sedentary time category and 30.1% in the high sedentary time category—than in the categories that met the recommendation—22.9% and 21.2%, respectively.

The prevalence of abdominal obesity also differed significantly across the categories (*p* = 0.001), with the highest prevalence observed in the high sedentary time/not meeting the physical activity recommendation category (39.5%). The prevalence of elevated blood pressure differed significantly across the categories (*p* < 0.001) and was highest in the low sedentary time/not meeting the recommendation category (51.9%), followed by the high sedentary time/not meeting the recommendation category (45.5%).

The prevalence of elevated fasting glucose also differed significantly across the categories (*p* < 0.001), with the highest prevalence in the low sedentary time/not meeting the recommendation category (44.3%) and the lowest prevalence in the high sedentary time/meeting the recommendation category (25.5%). The prevalence of elevated triglycerides did not differ significantly across the categories (*p* = 0.198). In contrast, the prevalence of reduced HDL-C differed significantly across the categories (*p* = 0.029), with the highest prevalence observed in the high sedentary time/not meeting the recommendation category (20.2%) (Table 2).

### 3.3. Association Between Joint Sedentary Time–Physical Activity Categories and Metabolic Syndrome

Complex-sample logistic regression analyses were conducted to examine the association between the joint sedentary time–physical activity categories and metabolic syndrome (Table 3). The low sedentary time/meeting the physical activity recommendation category was used as the reference group.

In the unadjusted model (Model 1), participants in the low sedentary time/not meeting the physical activity recommendation category had significantly higher odds of metabolic syndrome than the reference group (OR = 1.47, 95% CI: 1.08–1.99, *p* = 0.014). Significantly higher odds were also observed in the high sedentary time/not meeting the recommendation category (OR = 1.45, 95% CI: 1.15–1.83, *p* = 0.002). In contrast, the high sedentary time/meeting the recommendation category was not significantly associated with metabolic syndrome (OR = 0.91, 95% CI: 0.69–1.18, *p* = 0.462).

After adjustment for age and sex in Model 2, only the high sedentary time/not meeting the recommendation category remained significantly associated with higher odds of metabolic syndrome (OR = 1.36, 95% CI: 1.07–1.72, *p* = 0.012). The association for the low sedentary time/not meeting the recommendation category was no longer statistically significant (OR = 1.17, 95% CI: 0.86–1.60, *p* = 0.318). The high sedentary time/not meeting the recommendation category remained significantly associated with higher odds of metabolic syndrome after additional adjustment for education level and household income in Model 3 (OR = 1.42, 95% CI: 1.10–1.82, *p* = 0.007) and after further adjustment for smoking status, alcohol consumption, and average sleep duration in the primary adjusted model (Model 4) (OR = 1.38, 95% CI: 1.07–1.78, *p* = 0.013).

In the BMI-adjusted sensitivity analysis (Model 5), the point estimate for the high sedentary time/not meeting the recommendation category changed only modestly (OR = 1.34, 95% CI: 0.98–1.83, *p* = 0.065), although the confidence interval widened and the association was no longer statistically significant. Model 4 was therefore retained as the primary adjusted model, whereas Model 5 was interpreted only as a sensitivity analysis because of the potential for over-adjustment related to adiposity.

### 3.4. Independent Associations of Sedentary Time and Physical Activity with Metabolic Syndrome

Independent associations of sedentary time and physical activity with metabolic syndrome are presented in Table 4. In the mutually adjusted model including the covariates used in Model 4, high sedentary time (>8 h/day) was not significantly associated with metabolic syndrome compared with low sedentary time (≤8 h/day) (adjusted OR = 1.17, 95% CI: 0.96–1.43, *p* = 0.110). Participants who did not meet the physical activity recommendation had higher unadjusted odds of metabolic syndrome than those who met the recommendation (OR = 1.54, 95% CI: 1.26–1.88); however, the association was attenuated after mutual adjustment for sedentary time and the Model 4 covariates and was no longer statistically significant (adjusted OR = 1.19, 95% CI: 0.96–1.47, *p* = 0.109).

### 3.5. Interaction and Effect-Modification Analyses for Sedentary Time and Physical Activity in Relation to Metabolic Syndrome

The results of the interaction and effect-modification analyses are shown in Table 5. No statistically significant multiplicative interaction was observed between sedentary time and physical activity in relation to metabolic syndrome (interaction OR = 1.08, 95% CI: 0.74–1.57, *p* = 0.681). Similarly, the additive interaction indices did not indicate a significant interaction (RERI = 0.071, 95% CI: −0.199 to 0.341, *p* = 0.605; AP = 0.057, 95% CI: −0.163 to 0.277, *p* = 0.608; SI = 1.42, 95% CI: 0.25–7.96, *p* = 0.687).

In analyses stratified by physical activity recommendation status, high sedentary time was not significantly associated with metabolic syndrome among either participants who met the physical activity recommendation (OR = 1.07, 95% CI: 0.79–1.43, *p* = 0.663) or those who did not meet the recommendation (OR = 1.24, 95% CI: 0.96–1.61, *p* = 0.099). Furthermore, among participants with high sedentary time, the odds of metabolic syndrome did not differ significantly between those who did and did not meet the physical activity recommendation (OR = 1.23, 95% CI: 0.95–1.59, *p* = 0.116). These analyses provided no statistically significant evidence of effect modification by physical activity.

### 3.6. Sensitivity Analyses

Sensitivity analyses using alternative specifications of sedentary time and physical activity are presented in Supplementary Table S1. When sedentary time was modeled as a continuous variable, each additional hour per day of sedentary time was associated with 4% higher odds of metabolic syndrome after adjustment for physical activity recommendation status and the covariates included in Model 4 (adjusted OR = 1.04, 95% CI: 1.01–1.07, *p* = 0.011).

When total MVPA time was analyzed continuously per 150 min/week increase, the association with metabolic syndrome was not statistically significant (adjusted OR = 0.98, 95% CI: 0.93–1.04, *p* = 0.538). In the four-category analysis, compared with participants reporting no MVPA, those reporting 1–149 min/week (adjusted OR = 0.74, 95% CI: 0.59–0.93, *p* = 0.011) and ≥300 min/week (adjusted OR = 0.71, 95% CI: 0.54–0.93, *p* = 0.012) had significantly lower odds of metabolic syndrome, whereas the association for 150–299 min/week was not statistically significant (adjusted OR = 0.77, 95% CI: 0.58–1.03, *p* = 0.074).

In the sensitivity analysis additionally adjusted for BMI, the point estimate for the high sedentary time/not meeting the physical activity recommendation group remained similar to that in the primary adjusted model but was no longer statistically significant (adjusted OR = 1.34, 95% CI: 0.98–1.83, *p* = 0.065).

### 3.7. Comparison of Participants Included in and Excluded from the Final Analytic Sample

To assess potential selection bias, characteristics of participants included in the final analytic sample (*n* = 3075) were compared with those of participants excluded after the age-based eligibility criterion (*n* = 2958) (Supplementary Table S2). Participants included in the final analysis were younger than those excluded (49.02 vs. 50.85 years, *p* = 0.002), had a higher proportion of men (52.2% vs. 47.4%, *p* = 0.001), and reported slightly longer average sleep duration (7.47 vs. 7.37 h/day, *p* = 0.008). No statistically significant differences were observed in household income, educational attainment, smoking, alcohol drinking, high sedentary time, meeting the physical activity recommendation, or BMI. Notably, the distributions of the two primary exposure variables, sedentary time and physical activity, did not differ significantly between the included and excluded participants.

## 4. Discussion

This study examined the joint and independent associations of sedentary time and physical activity with metabolic syndrome and evaluated whether physical activity modified the association between sedentary time and metabolic syndrome among Korean adults. In the primary adjusted model (Model 4), participants with high sedentary time who did not meet the physical activity recommendation had 38% higher odds of metabolic syndrome than those with low sedentary time who met the recommendation. However, neither sedentary time nor physical activity was significantly associated with metabolic syndrome when examined as dichotomous exposures in mutually adjusted models. Furthermore, no statistically significant interaction between sedentary time and physical activity was observed on either the multiplicative or additive scale. In sensitivity analysis, each additional hour per day of sedentary time was associated with 4% higher odds of metabolic syndrome. Additional adjustment for BMI produced only a modest change in the point estimate for the high sedentary time/not meeting the recommendation group (OR, 1.38 to 1.34), although the confidence interval widened. Taken together, these findings indicate an association for the joint behavioral category but do not provide evidence that physical activity modifies or offsets the association between sedentary time and metabolic syndrome.

The findings of this study are generally in line with previous research examining how combined patterns of sedentary time and physical activity are associated with metabolic syndrome and other cardiometabolic outcomes. In a 10-year prospective Korean cohort, changes in sitting time and physical activity were jointly associated with incident metabolic syndrome. Participants who increased their physical activity had a lower incidence of metabolic syndrome than those whose sitting time increased and physical activity decreased [14]. Similarly, studies using accelerometer-based measures in Finnish adults have shown that combined patterns of sedentary time and physical activity are related to adiposity as well as lipid, glucose, and insulin biomarkers [12]. In Canadian adults, Rao et al. examined the independent and combined associations of sedentary time and MVPA with metabolic syndrome and explored whether MVPA modified the association between sedentary behavior and metabolic syndrome [15]. The term MVPA in that study refers to the physical activity exposure as defined by Rao et al., rather than to the specific MVPA metric constructed in the present analysis. Although patterns differed across MVPA levels, the moderating associations were not statistically significant [15]. This is consistent with the present study, in which neither multiplicative nor additive interaction between sedentary time and physical activity was statistically significant. Although the exposure measurements and outcome definitions differed across studies, these findings collectively support considering sedentary time and physical activity jointly in relation to metabolic health while distinguishing joint-category associations from formal statistical interaction.

Participants in the high sedentary time/meeting the physical activity recommendation category were younger and included higher proportions of men and ever-drinkers, as well as participants with a college education or higher and those in the highest household income quartile. These differences suggest that sociodemographic and behavioral characteristics may be associated with joint patterns of sedentary time and physical activity. The observed differences across the joint categories also underscore the importance of accounting for sociodemographic and health-related behavioral factors when examining their associations with metabolic syndrome. Future studies should examine how these characteristics are related to different sedentary time–physical activity patterns.

In the BMI-adjusted sensitivity analysis, the odds ratio for the high sedentary time/not meeting the physical activity recommendation category changed only modestly from 1.38 to 1.34, while the confidence interval widened and the *p*-value increased to 0.065. Therefore, the loss of statistical significance after BMI adjustment should not be interpreted as evidence that adiposity explains the observed association. BMI is closely related to waist circumference, which is itself a diagnostic component of metabolic syndrome, and may also lie on the causal pathway linking sedentary time and physical activity to metabolic syndrome. Accordingly, adjustment for BMI may represent over-adjustment rather than conventional confounding control. Previous studies have shown that sedentary behavior, physical inactivity, obesity, and metabolic syndrome are closely interrelated [8,9,22]. However, because the present study was cross-sectional and did not perform formal mediation analysis, it cannot determine whether adiposity mediates the association between sedentary time, physical activity, and metabolic syndrome. The BMI-adjusted model should therefore be interpreted as a sensitivity analysis rather than as evidence for a mediating or explanatory role of adiposity.

From a public health perspective, the present findings support considering sedentary time and physical activity together when evaluating behavioral patterns associated with metabolic syndrome. However, because no significant multiplicative or additive interaction was observed, the findings do not provide evidence that meeting the physical activity recommendation offsets the association between prolonged sedentary time and metabolic syndrome. Strategies aimed at reducing prolonged sedentary time and promoting sufficient physical activity may therefore be considered as complementary behavioral approaches rather than as substitutes for one another. Nevertheless, given the cross-sectional design of this study, the effectiveness of interventions targeting both behaviors cannot be inferred from the present findings. Prospective and intervention studies are needed to determine whether simultaneously reducing sedentary time and increasing physical activity improves metabolic health and to further clarify the role of adiposity in these relationships.

This study has several strengths. It used recent data from a nationally representative health survey, accounted for the complex sampling design of the KNHANES, and defined metabolic syndrome using clinically measured components. In addition to examining joint sedentary time–physical activity categories, the study evaluated the independent associations of sedentary time and physical activity and formally assessed interaction on both multiplicative and additive scales. Sensitivity analyses using continuous and alternative categorical specifications of sedentary time and physical activity further allowed for assessment of the robustness of the findings to exposure classification. These features enhance the relevance of the findings to the Korean adult population and provide a more comprehensive assessment of the relationships between sedentary time, physical activity, and metabolic syndrome.

This study has several limitations. First, because of its cross-sectional design, temporal and causal relationships among sedentary time, physical activity, and metabolic syndrome cannot be established. Second, sedentary time and physical activity were assessed using self-reported questionnaire data and may therefore be subject to recall error and exposure misclassification. In particular, the sedentary-time measure did not capture posture, breaks, or behavioral context, and the KNHANES physical activity questionnaire retained the ≥10 min bout criterion despite current WHO recommendations no longer requiring a minimum bout duration. However, sensitivity analyses using continuous and alternative categorical measures of sedentary time and physical activity were conducted to reduce concerns related to dichotomization. Third, although the distributions of sedentary time and physical activity did not differ significantly between included and excluded participants, some differences in demographic and behavioral characteristics were observed; therefore, selection bias cannot be excluded. Fourth, residual confounding may remain because factors such as dietary intake and occupational characteristics were not fully accounted for. Finally, because BMI is closely related to the abdominal obesity component of metabolic syndrome and may lie on the causal pathway, the BMI-adjusted model may be susceptible to over-adjustment and was therefore interpreted only as a sensitivity analysis.

## 5. Conclusions

Among Korean adults, the combination of high sedentary time and not meeting the physical activity recommendation was associated with higher odds of metabolic syndrome compared with low sedentary time and meeting the recommendation after adjustment for sociodemographic and health-related behavioral factors. However, no statistically significant multiplicative or additive interaction between sedentary time and physical activity was identified, and the findings do not support the interpretation that physical activity modifies or offsets the association between prolonged sedentary time and metabolic syndrome. Sensitivity analysis using sedentary time as a continuous variable further showed that longer sedentary time was associated with higher odds of metabolic syndrome. These findings support the joint consideration of sedentary time and physical activity when assessing metabolic health. Prospective studies using more detailed or objective measures of sedentary behavior and physical activity are needed to clarify their temporal, dose–response, and combined relationships with metabolic syndrome.

## Figures and Tables

**Figure 1:**
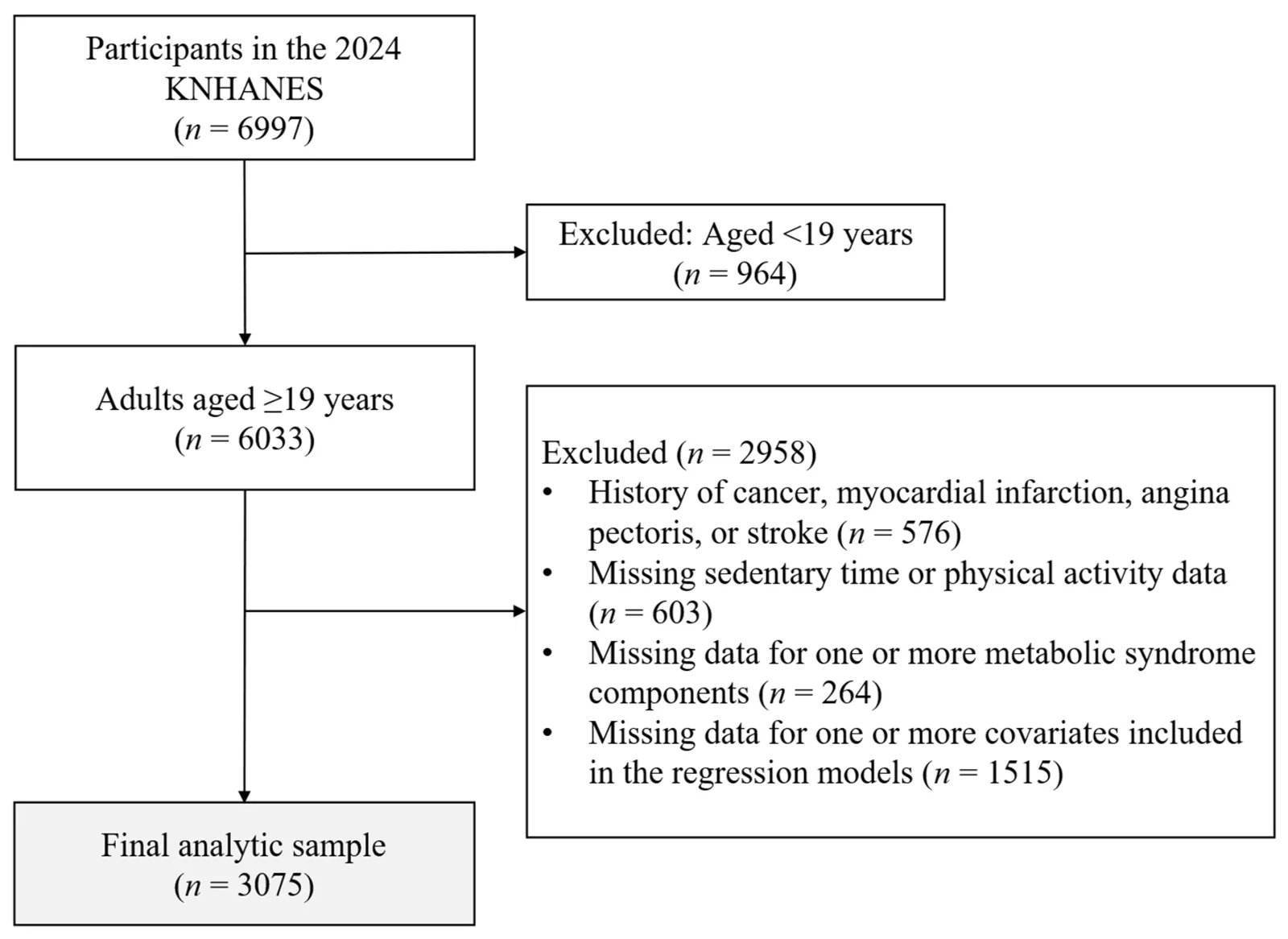
Flow diagram of participant selection from the 2024 Korea National Health and Nutrition Examination Survey.

**Table 1:** General characteristics of participants by sedentary time–physical activity groups (*n* = 3075).

Variable	Category	Total	Low ST + Meeting PA Recommendation (*n* = 652)	Low ST + Not Meeting PA Recommendation (*n* = 672)	High ST + Meeting PA Recommendation (*n* = 729)	High ST + Not Meeting PA Recommendation (*n* = 1022)	*p*
Age (years)		49.02 ± 0.60	48.13 ± 0.87	56.39 ± 0.77	41.58 ± 0.83	51.25 ± 0.81	<0.001
Sex	Men	1363 (52.2)	297 (53.8)	289 (48.2)	356 (57.1)	421 (49.3)	0.007
	Women	1712 (47.8)	355 (46.2)	383 (51.8)	373 (42.9)	601 (50.7)	
Education	Middle school or less	807 (18.6)	134 (14.9)	252 (29.3)	100 (9.3)	321 (22.3)	<0.001
	High school	820 (25.6)	215 (31.9)	223 (33.7)	143 (17.4)	239 (23.1)	
	College or university	1280 (49.8)	269 (48.2)	180 (34.1)	422 (64.5)	409 (48.4)	
	Graduate school	168 (6.0)	34 (5.0)	17 (2.9)	64 (8.8)	53 (6.2)	
Household income (quartile)	Q1 (lowest)	561 (14.4)	76 (9.3)	124 (14.8)	121 (13.5)	240 (18.5)	<0.001
	Q2	763 (23.5)	178 (26.5)	197 (27.7)	146 (18.2)	242 (23.3)	
	Q3	852 (29.7)	200 (33.2)	177 (27.9)	206 (29.5)	269 (28.6)	
	Q4 (highest)	899 (32.3)	198 (31.1)	174 (29.6)	256 (38.8)	271 (29.6)	
Smoking	Never-smoker	1907 (58.0)	405 (57.5)	406 (57.3)	449 (58.2)	647 (58.7)	0.960
	Ever-smoker	1168 (42.0)	247 (42.5)	266 (42.7)	280 (41.8)	375 (41.3)	
Alcohol drinking	Never-drinker	327 (8.0)	50 (6.2)	93 (10.9)	56 (5.6)	128 (9.5)	<0.001
	Ever-drinker	2748 (92.0)	602 (93.8)	579 (89.1)	673 (94.4)	894 (90.5)	
Sleep duration (h/day)		7.47 ± 0.02	7.65 ± 0.05	7.66 ± 0.06	7.29 ± 0.05	7.37 ± 0.04	0.013
BMI (kg/m^2^)		24.25 ± 0.10	24.34 ± 0.18	24.01 ± 0.16	24.38 ± 0.19	24.23 ± 0.17	0.440

Values are presented as unweighted frequencies with weighted percentages for categorical variables and as weighted means ± standard errors (SEs) for continuous variables. *p*-values were obtained using complex-sample general linear models for continuous variables and Rao–Scott adjusted chi-square tests for categorical variables. Abbreviations: ST, sedentary time; PA, physical activity; BMI, body mass index.

**Table 2:** Prevalence of metabolic syndrome and its components by sedentary time–physical activity groups (*n* = 3075).

Variable	Category	Total	Low ST + Meeting PA Recommendation (*n* = 652)	Low ST + Not Meeting PA Recommendation (*n* = 672)	High ST + Meeting PA Recommendation (*n* = 729)	High ST + Not Meeting PA Recommendation (*n* = 1022)	*p*
Metabolic syndrome	No	2219 (73.8)	500 (77.1)	454 (69.6)	572 (78.8)	693 (69.9)	<0.001
	Yes	856 (26.2)	152 (22.9)	218 (30.4)	157 (21.2)	329 (30.1)	
Abdominal obesity	No	1991 (65.4)	449 (67.1)	417 (64.1)	517 (70.9)	608 (60.5)	0.001
	Yes	1084 (34.6)	203 (32.9)	255 (35.9)	212 (29.1)	414 (39.5)	
Elevated blood pressure	No	1585 (56.2)	348 (56.8)	296 (48.1)	448 (63.7)	493 (54.5)	<0.001
	Yes	1490 (43.8)	304 (43.2)	376 (51.9)	281 (36.3)	529 (45.5)	
Elevated fasting glucose	No	1876 (64.8)	401 (64.0)	355 (55.7)	520 (74.5)	600 (63.0)	<0.001
	Yes	1199 (35.2)	251 (36.0)	317 (44.3)	209 (25.5)	422 (37.0)	
Elevated triglycerides	No	2335 (74.1)	509 (76.2)	494 (72.9)	573 (76.0)	759 (71.8)	0.198
	Yes	740 (25.9)	143 (23.8)	178 (27.1)	156 (24.0)	263 (28.2)	
Reduced HDL cholesterol	No	2514 (82.8)	555 (85.0)	549 (82.4)	617 (85.0)	793 (79.8)	0.029
	Yes	561 (17.2)	97 (15.0)	123 (17.6)	112 (15.0)	229 (20.2)	

Values are presented as unweighted frequencies with weighted percentages. *p*-values were obtained using Rao–Scott adjusted chi-square tests. Abbreviations: ST, sedentary time; PA, physical activity.

**Table 3:** Association between sedentary time–physical activity groups and metabolic syndrome.

Category	Metabolic Syndrome									
	Model 1		Model 2		Model 3		Model 4		Model 5	
	OR (95% CI)	*p*	OR (95% CI)	*p*	OR (95% CI)	*p*	OR (95% CI)	*p*	OR (95% CI)	*p*
Low ST + meeting PA recommendation (ref)	1 (ref)		1 (ref)		1 (ref)		1 (ref)		1 (ref)	
Low ST + not meeting PA recommendation	1.47 (1.08–1.99)	0.014	1.17 (0.86–1.60)	0.318	1.14 (0.84–1.56)	0.404	1.14 (0.83–1.55)	0.415	1.24 (0.87–1.77)	0.233
High ST + meeting PA recommendation	0.91 (0.69–1.18)	0.462	1.08 (0.82–1.44)	0.576	1.15 (0.86–1.54)	0.340	1.12 (0.84–1.50)	0.423	1.15 (0.81–1.62)	0.428
High ST + not meeting PA recommendation	1.45 (1.15–1.83)	0.002	1.36 (1.07–1.72)	0.012	1.42 (1.10–1.82)	0.007	1.38 (1.07–1.78)	0.013	1.34 (0.98–1.83)	0.065

Values are presented as odds ratios (ORs) with 95% confidence intervals (CIs). Abbreviations: ST, sedentary time; PA, physical activity. Model 1: Crude model (unadjusted). Model 2: Model 1 adjusted for age and sex. Model 3: Model 2 additionally adjusted for sociodemographic factors (education level and household income). Model 4: Model 3 additionally adjusted for health-related behaviors (smoking status, alcohol drinking, and average sleep duration). Model 5: Model 5 included 3048 participants because BMI data were missing for 27 participants. Model 4 was considered the primary adjusted model, whereas Model 5 was treated as a sensitivity analysis because of the potential for over-adjustment related to adiposity.

**Table 4:** Independent Associations of Sedentary Time and Physical Activity with Metabolic Syndrome in Mutually Adjusted Models.

Exposure	Category	*n*	Weighted MetS, %	Unadjusted OR (95% CI)	Adjusted OR (95% CI)	*p*
Sedentary time	≤8 h/day	1324	26.4	1 (ref)	1 (ref)	
	>8 h/day	1751	26.1	0.98 (0.83–1.17)	1.17 (0.96–1.43)	0.110
Physical activity	Meeting recommendation	1381	22.0	1 (ref)	1 (ref)	
	Not meeting recommendation	1694	30.2	1.54 (1.26–1.88)	1.19 (0.96–1.47)	0.109

*p*-values correspond to the fully adjusted model. Adjusted estimates mutually included sedentary time and physical activity and controlled for the covariates included in Model 4: age, sex, education level, household income, smoking status, alcohol consumption, and average sleep duration. Abbreviations: OR, odds ratio; CI, confidence interval; MetS, metabolic syndrome.

**Table 5:** Multiplicative and Additive Interaction Measures and Stratified Associations of Sedentary Time and Physical Activity with Metabolic Syndrome.

Analysis	Comparison/Index	Estimate	95% CI	*p*
Multiplicative interaction	High ST × not meeting PA recommendation	Interaction OR = 1.08	0.74–1.57	0.681
Additive interaction ^1^	RERI	0.071	−0.199 to 0.341	0.605
Additive interaction ^1^	AP	0.057	−0.163 to 0.277	0.608
Additive interaction ^1^	Synergy index (SI)	1.42	0.25–7.96	0.687
PA-stratified association	High vs. low ST among participants meeting PA recommendation	OR = 1.07	0.79–1.43	0.663
PA-stratified association	High vs. low ST among participants not meeting PA recommendation	OR = 1.24	0.96–1.61	0.099
Direct joint-category comparison	High ST/not meeting PA vs. high ST/meeting PA	OR = 1.23	0.95–1.59	0.116

All models were adjusted for age, sex, household income, education, smoking, alcohol drinking, and average sleep duration. The multiplicative interaction was tested on the logistic-regression scale. ^1^ Additive interaction indices were calculated from adjusted prevalence ratios estimated using survey-weighted modified Poisson regression because MetS was common in the study population. Abbreviations: RERI = relative excess risk due to interaction; AP = attributable proportion due to interaction; SI = synergy index; ST = sedentary time; PA = physical activity.

## Data Availability

The data analyzed in this study are publicly available from the Korea Disease Control and Prevention Agency through the KNHANES website.

## Supplementary Materials

The following supporting information can be downloaded at https://www.mdpi.com/article/10.3390/healthcare14193208/s1, Table S1: Sensitivity Analyses Using Alternative Exposure Specifications and Additional BMI Adjustment; Table S2: Comparison of Participants Included in and Excluded from the Final Analytic Sample.
