# Supplementary material for: Joint Association of Sedentary Time and Physical Activity with Metabolic Syndrome Among Korean Adults: A Cross-Sectional Analysis of the 2024 Korea National Health and Nutrition Examination Survey
Source: Healthcare (Basel). 2026 Sep 28;14(19):3208. doi: 10.3390/healthcare14193208 (PMC13665540; doi:10.3390/healthcare14193208)
Supplement: Supplementary file 1 [file healthcare-14-03208-s001.zip › healthcare-4517098-supplementary.pdf]

Supplementary Table S1. Sensitivity Analyses Using Alternative Exposure Specifications and Additional BMI Adjustment

Sensitivity analysis	Exposure / category	n	Adjusted OR	95% CI	*p*
Continuous sedentary time	Per 1 h/day increase in sedentary time	3,075	1.04	1.01–1.07	0.011
Continuous total physical activity	Per 150 min/week increase in total MVPA time	3,075	0.98	0.93–1.04	0.538
Total MVPA time, 4 categories	0 min/week	837	1 (ref)	—	—
	1–149 min/week	857	0.74	0.59–0.93	0.011
	150–299 min/week	601	0.77	0.58–1.03	0.074
	≥300 min/week	780	0.71	0.54–0.93	0.012
BMI-adjusted joint categories (Model 5)	Low ST + PA meeting	—	1 (ref)	—	—
	Low ST + PA not meeting	—	1.24	0.87–1.77	0.233
	High ST + PA meeting	—	1.15	0.81–1.62	0.428
	High ST + PA not meeting	—	1.34	0.98–1.83	0.065

The continuous sedentary-time model additionally adjusted for physical-activity recommendation status. The continuous and 4-category total MVPA time models adjusted for continuous sedentary time. Total MVPA time was calculated as the sum of weekly moderate- and vigorous-intensity activity minutes. All sensitivity analyses controlled for age, sex, household income, education, smoking, alcohol drinking, and average sleep duration. Model 5 additionally adjusted for BMI and included 3,048 participants because BMI was missing for 27 participants. Abbreviations: MVPA, moderate-to-vigorous physical activity; BMI, body mass index.

Supplementary Table S2. Comparison of Participants Included in and Excluded from the Final Analytic Sample

Characteristics	Included (n=3,075)	Excluded (n=2,958)	*p*
Age, years	49.02 ± 0.60	50.85 ± 0.49	0.002
Male, %	52.2	47.4	0.001
Highest household income quartile, %	32.3	33.4	0.488
College education or higher, %	55.8	56.6	0.587
Ever smoking, %	42.0	40.1	0.177
Ever drinking, %	92.0	92.8	0.270
Average sleep duration, h/day	7.47 ± 0.02	7.37 ± 0.03	0.008
High sedentary time (>8 h/day), %	58.4	60.1	0.259
Meeting physical activity recommendation, %	48.4	46.4	0.235
BMI, kg/m^2	24.25 ± 0.10	24.36 ± 0.09	0.374

Values are weighted means ± standard errors or weighted percentages. Analyses accounted for KNHANES sampling weights, strata, and primary sampling units. Available cases were used for each characteristic because some variables were missing among participants excluded from the final analysis.
